# Re: HPV vaccination for penile cancer eradication: cost effectiveness view

**DOI:** 10.1590/S1677-5538.IBJU.2018.0659

**Published:** 2019

**Authors:** Beuy Joob, Viroj Wiwanitkti

**Affiliations:** 1 Sanitation Medical Academic Center, Bangkok Thailand;; 2 DY Patil University, Pune, India

*To the editor,*

The contrasting opinions on using HPV vaccination for penile cancer eradication is a very interesting topic. Voris et al. noted that “it is recommended vaccination of adolescents (boys and girls) with 11-12 years old, or before the beginning of sexual life (preferably) (1) whereas Ornellas et al. noted that “the male public HPV vaccination is a good measure to prevent not just cervical cancer but some head and neck cancers, some penile warts and the majority of anal cancers. However, it is not clear if it will be enough to eradicate or reduce the prevalence of penile cancer (2). In fact, there are many considerations before the implementation of any new cancer vaccines. Effectiveness and safety are the basic focused concerns. Nevertheless, an additional consideration for using as public health policies that affect mass of populations is usually on the medical economics issue. We would like to share ideas on this issue based on the situation in our setting, a country in Indochina.

In our setting, the incidence of penile cancer is not high and significant lower contrasting to the incidence of cervix cancer. Based on the statistics of Thai National Cancer Institute, the annual incidence of HPV related cervix cancer is nearly 500.000 cases whereas the incidence of HPV related penile cancer is less than 10.000 cases. Although HPV cervix cancer vaccination has already been implemented as national free vaccination policies, there has never been proven evidence that the HPV penile cancer vaccination is cost effective. Based on the significant discrepancy of cancer incidence, the cost per utility (cancer case prevention) is significant higher, up to 50 times, in HPV penile cancer vaccination. Therefore, the HPV penile cancer vaccination has never been recommended in our country. Therefore, to implement HPV penile cancer vaccination or not in any setting, an additional factor to be thought is on the cost effectiveness.
